# The Neurological Implications of COVID-19: A Comprehensive Narrative Review

**DOI:** 10.7759/cureus.60376

**Published:** 2024-05-15

**Authors:** Ithamar Cheyne, Venmanassery Sreejan Gopinath, Neeharika Muppa, Angel Emanuel Armas, Maria Sophia Gil Agurto, Sai Abhigna Akula, Shubhangi Nagpal, Muhammad Sheraz Yousaf, Ali Haider

**Affiliations:** 1 Critical Care, Medical University of Warsaw, Warsaw, POL; 2 Internal Medicine, University Hospital Coventry and Warwickshire, Coventry, GBR; 3 School of Medicine, St. George’s University, St. George’s, GRD; 4 Internal Medicine, Cardiac Arrhythmia Service, Harvard Medical School, Boston, USA; 5 Internal Medicine, School of Medicine, San Martin de Porres University, Lima, PER; 6 Internal Medicine, School of Medicine, St. George's University, St. George's, GRD; 7 Internal Medicine, Guru Gobind Singh Government Hospital, New Delhi, IND; 8 Internal Medicine, Medstar Health, Baltimore, USA; 9 Allied Health Sciences, The University of Lahore, Gujrat Campus, Gujrat, PAK

**Keywords:** guillain-barre syndrome, encephalitis, parkinson's disease, neuroinvasion, neuroimmunology, treatment strategies, neuropathogenesis, neurological complications, sars-cov-2, covid-19

## Abstract

The COVID-19 pandemic caused by the coronavirus SARS-CoV-2 revealed a huge number of problems as well as discoveries in medicine, notably, regarding the effects of the virus on the central nervous system (CNS) and peripheral nervous system (PNS). This paper is a narrative review that takes a deep dive into the complex interactions between COVID-19 and the NS. Therefore, this paper explains the broad range of neurological manifestations and neurodegenerative diseases caused by the virus. It carefully considers the routes through which SARS-CoV-2 reaches the NS, including the olfactory system and of course, the hematogenous route, which are also covered when discussing the virus's direct and indirect mechanisms of neuropathogenesis. Besides neurological pathologies such as stroke, encephalitis, Guillain-Barré syndrome, Parkinson’s disease, and multiple sclerosis, the focus area is also given to the challenges of making diagnosis, treatment, and management of these conditions during the pandemic. The review also examines the strategic and interventional approaches utilized to prevent these disorders, as well as the ACE2 receptors implicated in the mediation of neurological effects caused by COVID-19. This detailed overview, which combines research outputs with case data, is directed at tackling this pandemic challenge, with a view toward better patient care and outcomes in the future.

## Introduction and background

The COVID-19 pandemic has significantly affected the world and has immediate health implications [1]. Among the numerous complications of COVID-19 caused by the severe acute respiratory syndrome coronavirus 2 (SARS-CoV-2), its effects on the central nervous system (CNS) have been a concern to the scientific community, particularly in people with existing neurological conditions [2-4]. These presentations range from headache and anosmia to severe cases such as encephalitis, stroke, and acute disseminated encephalomyelitis (ADEM). Besides, while respiratory manifestations of COVID-19 often dominate clinical talks [5], their impact on morbidity, mortality, and long-term quality of life has highlighted an increasing emphasis on neurologic sequelae [6,7]. In this context of COVID-19, vulnerable populations like patients suffering from pre-existing CNS disorders, including multiple sclerosis (MS), Parkinson’s disease (PD), epilepsy, and dementia, among others, face unique challenges [8,9]. This narrative review examines the long-term neurological consequences and complications of COVID-19 in such vulnerable populations. Moreover, the connection between brain health and virus treatment issues is illuminated.

The heterogeneity of neurological manifestations observed in COVID-19 has underscored the need for multidisciplinary patient care that draws expertise from neurology, infectious diseases, immunology, and critical care medicine [7]. Even though there is growing acknowledgment of the fact that COVID-19 can affect the brain, significant knowledge gaps still exist regarding long-term neurological complications and how best we can deal with them, especially in patients with pre-existing CNS disorders. Limited information exists about COVID-19 progression in people with comorbidities related to their nervous systems, such as disability accumulation [10]. The novelty of this review lies in its comprehensive exploration of the long-term neurological implications and complications of COVID-19 in patients with pre-existing CNS disorders, as well as its treatment and outcomes in these individuals. The COVID-19 pandemic has brought about unprecedented challenges for medical professionals worldwide and has highlighted the importance of understanding the potential neurological consequences of this novel virus [5,11].

Therefore, this review aims to summarize available literature to fill these gaps by exploring the long-term neurologic effects of COVID-19 on individuals who already had CNS disorders and those without a prior history of the disease, leading to de novo neurological complications. The current study aims to influence clinical practice by critically appraising existing research, identifying areas for further investigation, and eventually helping guide management decisions, thereby improving outcomes for patients suffering from COVID-19 and neurological illnesses.

## Review

Mode of entry of COVID-19 into CNS

Structural Composition of SARS-CoV-2 and Neuroinvasion

Previous reports have shown that SARS-CoV-2 can enter the CNS, resulting in various disorders [12-14]. The ability of SARS-CoV-2 to invade the CNS raises questions about the routes of neuroinvasion and the underlying mechanisms. It is important to identify all routes of entry of the virus into the host in order to prevent the spread of SARS-CoV-2. Moreover, this will help develop treatment methods such as virus attachment inhibitors, neutralizing antibodies, and vaccines to avoid the effects of COVID-19 on neurocognition and on patients with preexisting neurological diseases.

SARS-CoV-2 is a single-stranded positive-sense RNA virus that contains four structural proteins: N, M, E, and S. Its envelope is mainly composed of spike glycoprotein (S protein), which is composed of two subunits: S1 and S2 [15]. The S1 subunit contains the receptor-binding domain RBD that interacts with ACE2, which is a membrane receptor on host cells. The S2 subunit contains the transmembrane domain (TD) and is anchored to the viral membrane [16,17]. Studies revealed the trimeric structure of the S protein and its conformational changes during viral attachment and entry to host cells. S protein is assembled as a homotrimer and inserted in multiple copies into the virion membrane, giving it its crown-like appearance [18,19].

The S protein must undergo cleavage by proteases at the appropriate position to accomplish its fusion function [20]; it is cleaved by proprotein convertases such as the transmembrane protease serine 2 (TMPRSS2) and furin in the virus-producer cells. Recent studies have demonstrated that several other cellular proteases besides TMPRSS2 and furin can induce proteolytic cleavage of the S protein, such as TMPRSS4, trypsin-like proteases, and cathepsin L [21]. A hypothesis about a novel concept states that the cleavage of the S protein S1 subunit by furin results in a higher number of free S protein molecules, even with higher affinity than the original one, that can bind other ACE2 and other receptors in various tissues. This hypothesis suggests that this mechanism is responsible for the effects of COVID-19 on the brain, and if it becomes proven, furin inhibitors and vaccines may benefit patients with neurological diseases due to COVID-19 [16].

As with other members of the coronavirus family, it is known that angiotensin-converting enzyme 2 (ACE2) has a high affinity for the S protein from SARS-CoV-2 [22-25]. Differences in host susceptibility arise from the interaction between the receptor-binding domain and ACE2, while discrepancies in viral tropism and tissue distribution are likely determined by the distribution and abundance of ACE2 and TMPRSS2 and possibly other cellular proteases that are still under investigation [21]. To date, there are several studies focusing entirely on ACE2 as the major SARS-CoV-2 entry host receptor field [4], but research also shows ACE2 is expressed at very low levels in brain tissue, raising the question about other cofactors implicated in virus-host cell interactions in cells with low ACE2 expression [26].

It is now established in many studies that the entry of COVID-19 into human cells requires not only the presence of ACE2 but additional receptors and cofactors [27]. For example, there is evidence that shows another host factor for SARS-CoV-2 called neuropilin-1 (NRP1) that can potentiate infectivity. The study concluded that although NRP1 did not promote infection itself, its coexpression with ACE2 and TMPRSS2 enhanced infection [26,28]. Further studies should be conducted to study the distribution and abundance of these host factors in brain tissue to determine neurotropism.

Over the years since COVID-19 appeared, several studies focused on looking for neurotropism using different viral probing techniques in post-mortem patients, in vivo (animals), and in vitro stem cell-derived human neuronal models, many of them with contradictory outcomes. Recent research has shown that the neuropathogenesis of COVID-19 is not only caused by direct neuronal infection. A recent study tested infection and replication between neurons of SARS-CoV-2 in induced pluripotent stem cell-derived humans, where infection in just a few neurons was observed. Hence, they suggested that there are also indirect mechanisms responsible for the reported neurological complications from COVID-19 that should be investigated, such as immune-mediated neuroinflammation due to invasion of non-neuronal cells such as microglia and astrocytes or neurovascular thrombosis due to a hypercoagulable state [14,29-31].

The long-term outcomes of neurological manifestations after SARS-CoV-2 infection are still being investigated. Besides, it remains unknown how far the damage caused by COVID-19 in the nervous system goes or if neurological manifestations are attributable to secondary mechanisms. This raises the question of direct infection of CNS neurons that might explain neurological complications.

Olfactory System Pathway Via Transcribrial Route

The idea that viruses use the olfactory nerve route to reach the brain is not new. Various studies have indicated the olfactory sensory epithelium as a possible direct SARS-CoV-2 access to the brain. Reports at the beginning of the pandemic suggested an association between COVID-19 and anosmia [32], describing this symptom as a biomarker and a predictor of COVID-19 infection [33-35].

A first report about a patient with COVID-19 infection and anosmia described an MRI showing bilateral hyperintensity of the posterior gyrus rectus of the cortical region and the olfactory bulbs, suggesting viral involvement in an area of the brain associated with olfaction [36]. Preliminary findings were against this theory; some authors looked for expression of ACE2 and TMPRSS2 first in mice [37], and then in human olfactory sensory tissue by collecting biopsies via nasal endoscopic surgery; the results demonstrated that olfactory sustentacular cells expressed ACE2 and TMPRSS2, rather than olfactory sensory neurons suggesting that neurological manifestations as anosmia are due to inflammation of non-neuronal cell types in the olfactory epithelium [38]. In a group of COVID-19 deceased patients, it was observed that the parenchyma of the olfactory bulb remained unaffected, indicating no evidence of infection in olfactory sensory neurons [39].

On the other hand, several methods are reported in the literature to address this issue [40]. Another study provides proof of SARS-CoV-2 infection into the CNS by transmucosal invasion via regional nervous structures detecting the virus RNA in autopsy material from 33 deceased patients with COVID-19 infection [41]. Furthermore, another experimental research on rhesus monkeys that were inoculated with SARS-CoV-2 intranasally resulted in CNS infection, with virus protein observed not only in neurons but also in astrocytes and microglia of monkeys. However, the research suggested that SARS-CoV-2 may use alternative receptors besides ACE2 since they found low mRNA levels of ACE2 in the CNS [42]. A recent study examined alterations in the olfactory mucosa of rhesus macaque; several studies suggest that this species is chosen for their similarity to humans in nasal anatomy and immune response. Viral RNA was detected in the olfactory bulbs and neurons, particularly at three- and seven days post-infection, suggesting that SARS-CoV-2 infects the CNS through the olfactory system in the initial phase of infection [43].

However, much of the existing literature had problems with the various methods available for SARS-CoV-2 detection in different tissues, leading to misinterpretation [44]. Consequently, several studies agree that the primary target of SARS-CoV-2 in the olfactory neuroepithelium is sustentacular cells, but a direct infection of olfactory sensory neurons was not evidenced or rarely observed, thus making neuroinvasion via olfactory pathway unlikely. Additional studies to further understand this issue are required.

Neurons Retrograde Across Synapses

Retrograde axonal transport may also be another potential route for COVID-19 to enter the brain via other cranial nerves such as the vagus and trigeminal nerves. Besides the olfactory pathway, COVID-19 may also use the trigeminal nerve to enter the brain, which projects to the walls of the nasal passages [45].

**Figure 1:**
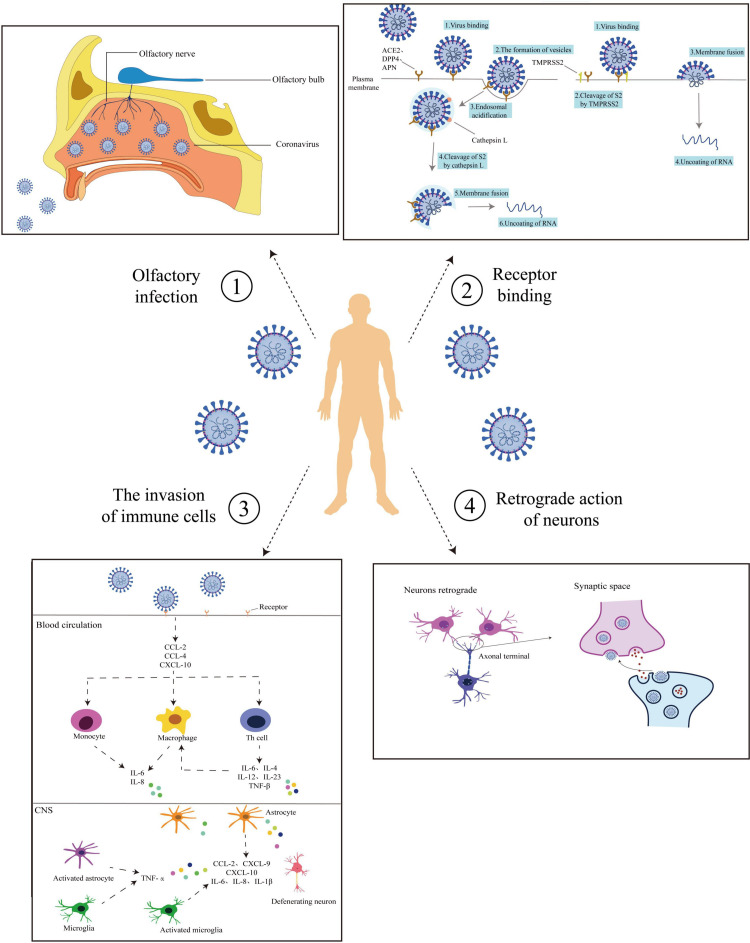
Routes of Coronavirus Infection into the CNS (1) Olfactory pathway: The virus enters via the mouth, binding to olfactory nerve receptors, and transmitting through the nerve to the brain. (2) receptor binding: The virus binds host cell receptors with TMPRSS2 aid, entering via endocytosis or fusing with cell membranes for nucleic acid release. (3) Trojan Horse mechanism: The virus activates pro-inflammatory pathways via ACE2 and NF-κB, inducing a cytokine storm and immune cell recruitment, potentially damaging the blood-brain barrier and activating neurotoxic responses. (4) Neurons retrograde: The virus spreads retro-transsynaptically after infecting peripheral neurons, reaching central or other peripheral neurons. CNS: central nervous system; TMPRSS2: transmembrane protease serine 2; ACE2: angiotensin-converting enzyme 2; NF-κB: nuclear factor kappa-light-chain-enhancer of activated B Cells Reproduced under the terms and conditions of the Creative Commons Attribution License (CC-BY) from Ref [47]. Copyright © 2023 Sha and Chen. Published by Frontiers in Neuroscience.

It is known that many viruses affect the gastrointestinal tract and the enteric nervous system as rotavirus. Jiao et al. explored SARS-CoV-2 neuroinvasion on rhesus monkeys, suggesting that the gastrointestinal (GI) tract may also be a mode of entry [42]. ACE2 receptor and TMPRSS2 are highly expressed in GI cells, particularly enterochromaffin cells that also express NRP1, suggesting that enteric glial cells and neurons infected with SARS-CoV-2 work as an axonal retrograde transport route to the CNS via the vagus nerve transmitting the infection to the brainstem vagal centers (nucleus tractus solitaries) (Figure 1) [46].

A recently published study suggested that SARS-CoV-2 may invade the nervous system through the nervus terminalis, a group of sensory and autonomic nerve fibers that supply innervation to the Bowman glands in the olfactory epithelium and the vasculature around. Nervus terminalis expresses ACE2, cathepsin L, and B, reaching the brain through axon projections, particularly the hypothalamus [48]. However, research on this postulation remains limited. 

Hematogenous Spread

One of the most important biological barriers of the brain includes the blood-brain barrier (BBB), which separates blood from the fluid inside the brain and protects it from an entry of pathogens, neurotoxic debris, and potential neurotransmitters not suitable for neural function. However, neuroinvasive viruses such as SARS-CoV-2 change the BBB’s functionality due to specific axonal trafficking and transsynaptic transmission mechanisms that travel along nerve pathways and infect the brain [49]. There are several studies suggesting that the BBB breakdown and subsequent penetration into CNS are responsible for diverse neurological manifestations, but most of the studies are limited by a small sample size [50-52]. Many studies also demonstrate that long-term COVID-19 associated with neurological impairment, also known as brain fog, is secondary to a BBB disruption and persistent inflammation [51]. Anosmia is a major neurological symptom reported in mild and severe COVID-19 infection, and its mechanism is still being studied. A cohort study suggests that BBB dysfunction was not evident in patients with anosmia without cognitive decline, so this could indicate that it may not be a primary contributor to this symptom. However, the research did find a notable association between BBB disruption in the thalamus and the duration of anosmia [51].

Immune and Autoimmune Mechanisms

Many nervous system disorders are identified as post-infectious sequelae of COVID-19 where a direct infection of the nervous system was not proved with the isolation of virus or viral particles in the brain or cerebrospinal fluid, pointing toward the persistent immune activation and inflammation within the CNS and autoimmune mechanisms [53]. Close relations between various neuroimmune disorders affecting both central (limbic and brainstem encephalitis, acute disseminated encephalomyelitis [ADEM], and myelitis) and peripheral nervous systems (Guillain-Barré and Miller Fisher syndrome) were identified, either with or without the presence of autoantibodies, and showing response to immunotherapies [54]. Both innate and adaptive immune mechanisms through glial cells, endothelial cells, immune cells such as macrophages, CD8+ T cells, NK cells, neutrophils, and monocytes are known to be involved in the neuropathogenesis in patients with COVID-19 infection [55]. Demonstration of high frequencies of anti-neuronal antibodies in serum or CSF, in the absence of direct presence of COVID-19 viral particles in the brain, suggested autoimmune mechanisms and a causal link with neurological manifestations [56].

A systematic review suggested a close association between CNS demyelination and COVID-19 infection, with a suggestion of causal association [57]. Another systematic review on Guillain-Barré syndrome (GBS) in COVID-19 patients evaluated 109 cases and concluded the peripheral nervous system involvement in the form of sensorimotor demyelination and facial palsy showcasing a temporal association between the infection and the development of demyelination-related symptoms [58]. Many studies have also demonstrated other autoimmune disorders such as e vasculitis and arthritis [59], autoimmune encephalitis [60,61], myasthenia gravis, neuromyelitis optica spectrum disorder (NMOSD) [62], and autoimmune autonomic nervous system imbalance [63], showing molecular mimicry and consequent para and post-infectious immune activation as a mode of neuropathogenesis of COVID-19 even in the absence of direct neuroinvasion.

The relationship between COVID-19 and neurological pathologies

Stroke

Although the clinical symptoms associated with COVID-19 are primarily respiratory, increased occurrence of ischemic stroke was reported in patients with concurrent COVID-19 infection. Luo et al. report that the incidence rate of COVID-19-associated ischemic stroke is 2%, with predominance in males [64]. Hypertension (HTN), hyperlipidemia, and diabetes mellitus (DM) are high-risk factors for ischemic stroke in patients with COVID-19 infection, but with different prevalence when compared to ischemic stroke patients without COVID-19. Yaghi et al. found a lower incidence of HTN in patients with simultaneous ischemic stroke and COVID-19, while Luo et al. suggest a higher incidence of hyperlipidemia in such patients [64,65]. Some evidence suggests an increased incidence of early-onset stroke in patients infected with COVID-19, even without comorbidities, presenting only with mild respiratory symptoms; this suggests that all early-age stroke patients should be thoroughly investigated for COVID-19 infection [66].

Several possible neuro-pathomechanisms were suggested to explain the increased rate of incidence of ischemic stroke in COVID-19 patients. Qin et al. suggest that hypercoagulability and endothelial damage are results of cytokine storm, induced by the COVID-19 infection. Several articles manifest the importance of the ACE2 receptor as the key to understanding the increased risk of ischemic stroke incidence in COVID-19 patients. ACE2 receptors are abundant in the nervous system, especially in the brainstem and regions controlling the cardiovascular system, and may cause thrombosis, vasoconstriction, and hypertension, all of which might lead to ischemic stroke [67,68]. Although those are the two general approaches up to date, other pathomechanisms cannot be excluded.

COVID-19-associated ischemic stroke has unique manifestations and a course of disease. Contrary to ischemic stroke without COVID-19 infection, ischemic stroke in the presence of COVID-19 infection is associated with higher rates of cryptogenic subtype of ischemic stroke and lower incidence of small-vessel disease-related subtype of ischemic stroke [64]. A possible explanation for the high incidence of cryptogenic stroke is an incomplete workup of possible etiologies due to the overload of the healthcare system during the COVID-19 pandemic. Another unique feature of ischemic stroke during COVID-19 infection is more large vessel occlusions of the anterior circulation compared to the control group [68,69].

Encephalitis

Encephalitis is an inflammation of the brain tissue, resulting in neurological dysfunction. The most frequent etiology of encephalitis is viruses, although other etiologies, infectious and non-infectious, are abundantly described. The clinical manifestations of encephalitis are confusion, reduced consciousness, fever, headache, and seizures; its diagnosis is clinical, laboratory, and imaging-based [70]. The first case of COVID-19-induced encephalitis was described in February 2020 [12,71], and ever since, an increasing number of cases have been described, reaching 2.2% of all COVID-19 patients according to a Spanish study [71].

Direct invasion, systemic inflammation, and molecular mimicry are the general pathomechanisms of COVID-19-associated encephalitis. Direct COVID-19 invasion has two main mechanisms: transsynaptic propagation of ACE2 receptors via the olfactory route and lymphocytic (especially monocytic) infection with COVID-19 and hematogenous transmission [72]. COVID-19-associated monocytic encephalitis (CAME) is septic perivascular and parenchymal inflammation due to monocyte activation and microglial hyperactivity. In CAME, the inflammatory pathways are IKK complex, TLR1/TLR2 cascade, NFkB phosphorylation, and JAK-STAT [73]. The inflammatory process damages pericytes, endothelial cells, and glial membranes. The most common locations of such damage are the brainstem, parietal lobe, hippocampus, and temporal lobe. A direct correlation between the degree of brain edema and the degree of astrocyte involvement was described [74]. This mechanism is unlikely to be the leading cause of encephalitis during COVID-19 infection due to CSF yielding negative results for COVID-19 in most patients undergoing chain-polymerase reaction testing [75].

Systemic inflammation induced by acute COVID-19 infection results in a cytokine storm and systemic inflammatory response syndrome. The pro-inflammatory molecules are transmitted throughout the body, resulting in a generalized pro-inflammatory state that attacks different body organs, including the brain [76].

Although an uncommon complication, encephalitis with the onset of COVID-19 increases mortality by a factor of four, with an elevation of 3.4% mortality to general COVID-19 patients to an astonishing 13.4% in patients with encephalitis as a complication of COVID-19 infection [77]. Further investigation of the different pathomechanisms leading to encephalitis as a complication of COVID-19 infection is required, potentially leading to therapeutic developments or expenditure of the indications of already existing therapeutics.

GBS

Emerging evidence suggests a potential correlation between COVID-19 and GBS. GBS is an immune-mediated syndrome that appears after infections damaging peripheral nerves and nerve roots [78]. The general pathomechanism that results in GBS after COVID-19 infection is hyperactivity of the immune system, which results in autoimmunity against axonal proteins, leading to nerve cell damage and destruction. GBS can be subdivided into three major subtypes based on clinical findings and electrophysiological activity: acute motor sensory axonal neuropathy (AMSAN), acute motor axonal neuropathy (AMAN), and acute inflammatory demyelinating polyradiculoneuropathy (AIDP) [79]. Several infectious pathogens have been associated with GBS, including Epstein-Barr virus (EBV), Zika virus, *Campylobacter jejuni*, and more [80].

The damage is mediated by molecular mimicry, in which post-infectious antibodies (Ab) attack proteins present in axonal membranes. The most common antibodies described in the literature are anti-GD1b IgG and anti-ganglioside Ab [81]. Miller-Fisher syndrome (MFS) is a rare subtype of GBS presenting with ophthalmoplegia, ataxia, and areflexia and is usually associated with anti-GQ1B Ab.

Parkinson's Disease

Parkinson's disease (PD) is a slow-onset progressive neurodegenerative disorder usually present in the later stage of life, resulting in generalized bradykinesia and resting tremor or rigidity. The general pathomechanism of PD is an accumulation of alpha-synuclein mainly in the substantia nigra, leading to loss of dopamine in the basal ganglia, resulting in movement disorders [82]. The literature increasingly indicates that PD may serve as a predisposing factor for more severe COVID-19 infection and heightened mortality rates. Given that both PD and susceptibility to COVID-19 increase with age, there is a compelling need to investigate the correlation between the two conditions [82].

COVID-19 infection caused worsening in daily experiences, motor-related disabilities, and motor performance in PD patients. In addition, worsening was observed in levodopa-related motor responses and an increase in the "off "daily period (on-off phenomenon). This could be explained by either a systemic inflammatory response or by altered pharmacokinetics of levodopa [83]. The rest of the non-motor symptoms experienced by PD patients during COVID-19 infection could be attributed to the infection itself and, so far, have not been related solely to PD [83].

Multiple Sclerosis

Multiple sclerosis (MS) is an autoimmune inflammatory and demyelinating disease of the CNS. Damage to the myelin sheets results in various neurological disturbances such as vision loss, loss of coordination, spasms, muscle weakness, and many others. MS is a multifactorial disease influenced by genetic predisposition and environmental factors, including viral infections and vitamin D levels. There are four main phenotypes for MS: clinically isolated syndrome, relapsing-remitting MS (RRMS), secondary progressive MS (SPMS), and primary progressive MS (PPMS). RRMS is the most common phenotype affecting about 85% of patients with MS [84]. Several cases of immunopathogenesis leading to MS are described in the literature, for example, the activation of myelin-specific lymphocytes in peripheral lymphoid organs. It is induced by several factors, including recognition of microbial epitopes and molecular mimicry [85], which are associated with COVID-19 infection. T cell licensing is a newly proposed, genetic-predisposed, immunopathogenic process in which T cells in the lungs and spleen become pathogenic, migrate to the CNS, and facilitate BBB inflammation [86]. Another suggested immunopathogenesis is Th17 cell expansion in the gut, primarily due to the microbiota population [87]. Th17 cells are critical in opening the BBB and possess neurodegenerative properties [87]. Given that specific dysregulated immune pathways observed during severe COVID-19 overlap with immune abnormalities seen in MS, there is speculation that COVID-19 might serve as a risk factor for initiating or exacerbating MS in susceptible individuals [88]. Another proposed pathomechanism correlating COVID-19 and MS is the dysregulation of vitamin D. This pathomechanism has been discussed and investigated thoroughly but has not been established yet [88].

Th1-Th17 axis results in the production of INF-y and IL-17 (interleukin-17), associated with both COVID-19 and MS [89]. As discussed earlier, Th17 is produced in the gut and highly regulated by the microbiota. COVID-19 infection is associated with high levels of Th17, gut dysbiosis, and INF-y [90]. Those coincidental findings could lead to speculation of increased risk of aggravation of COVID-19 or MS in patients with MS or COVID-19, respectively [88]. Another possible immunopathogenesis correlating COVID-19 and MS is the inflammasome cascade. The inflammasome is a complex molecular platform activated by PAMPs and DAMPs, resulting in inflammatory reactions. NLRP3 (NOD-, LRR- and pyrin domain-containing protein 3) is an intracellular sensor, part of the inflammasome cascade, and highly associated with different stages of MS [91,92]. COVID-19 also activated the inflammasome and NLRP3, potentially providing another immunopathogenic correlation between MS and COVID-19 [93].

**Figure 2:**
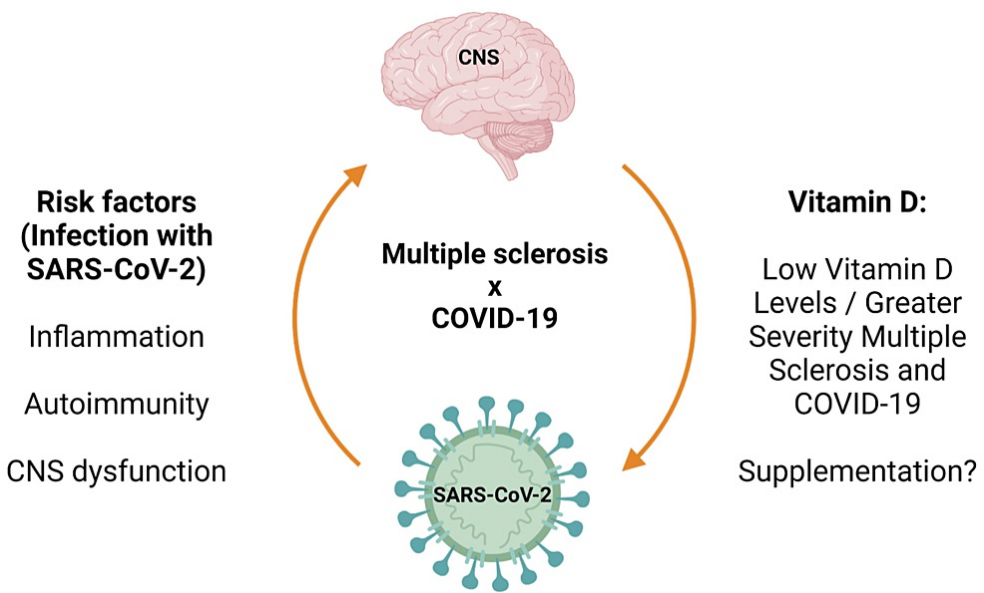
Main Effects of the COVID-19 Infection on Multiple Sclerosis SARS-CoV-2: severe acute respiratory syndrome coronavirus 2; CNS: central nervous system Reproduced under the terms and conditions of the Creative Commons Attribution (CC-BY) license from Ref. [88]. Copyright © 2023 by the authors. Licensee MDPI, Basel, Switzerland.

Despite all the pathogenic correlations between COVID-19 and MS, retrospective studies concluded that COVID-19 is not associated with an increased risk of MS relapse right after the infection [94]. Several other studies are mandated, such as a correlation between COVID-19 infection and MS relapse later, in the post-COVID infection period, and a correlation between the severity of either COVID-19 or MS in the presence of the other (Figure 2).

Neuropathic Pain

**Figure 3:**
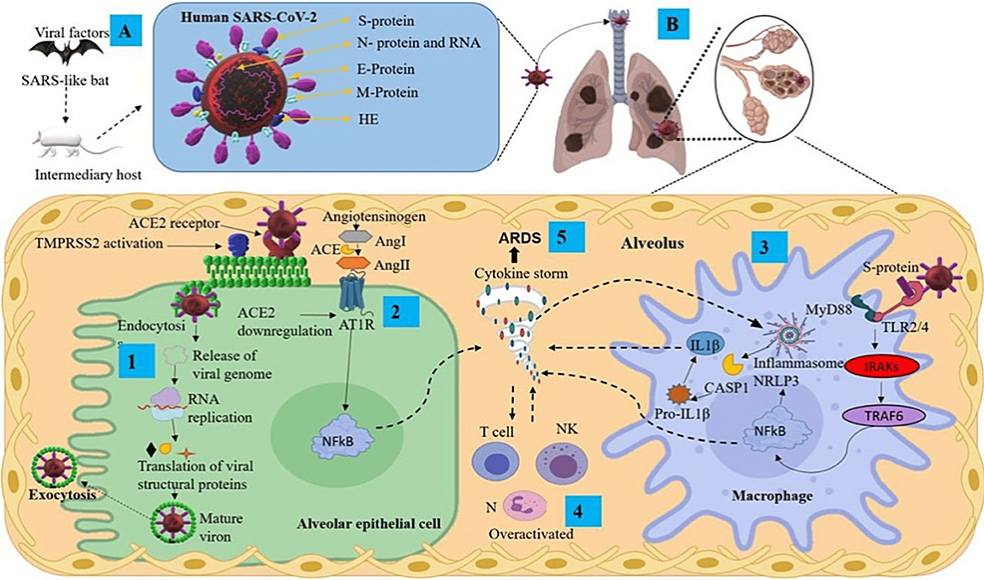
(A) Transmission and structural proteins of SARS-CoV-2. (B) Infection mechanisms and inflammatory response in the lungs, featuring key components ACE2: angiotensin-converting enzyme 2; Ang: angiotensin; ARDS: acute respiratory distress syndrome; AT1R: angiotensin II type I receptor; CASP1: caspase 1; E protein: envelope small membrane protein; HE: hemagglutinin esterase; IL1β: interleukin 1 beta; IRAKs: interleukin-1 receptor-associated kinases; M protein: membrane protein; MyD88: myeloid differentiation primary response 88; N protein: nucleoprotein; N: neutrophils; NF-κB: nuclear factor kappa B; NK: natural killer cells; NLRP3: nucleotide-binding domain; leucine-rich repeat-containing receptor; pyrin domain-containing 3; RNA: ribonucleic acid; S protein: spike protein; TMPRSS2: transmembrane serine protease 2; TRAF6: tumor necrosis factor receptor-associated factor 6 Reproduced under the terms and conditions of the Creative Commons Attribution (CC-BY) license from Ref. [96]. Copyright © 2021 Pacheco-Herrero, Soto-Rojas, Harrington, et al. Published by Frontiers in Neurology.

Pain is one of the most common complaints associated with COVID-19 infection. In the acute phase, headaches and arthralgias are common symptoms. During long COVID syndrome, neuropathic pain affects one out of three patients, bringing the prevalence of neuropathic pain to one out of nine patients infected with COVID-19 in general. While many symptoms affiliated with long COVID are well investigated, increased nociceptive stimulation that results in neuropathic pain is one of the least investigated ones (Figure 3A-3B) [95].

The lack of scientific evidence correlating neuropathic pain and COVID-19 infection is surprising, given the high incidence of neuropathic pain after or during COVID-19 infection. Further investigation of different pathomechanisms and clinical associations is required.

Myasthenia Gravis

Infection with COVID-19 is associated with several neurological diseases caused by autoimmune antibodies. One of the most prominent associations is with myasthenia gravis (MG) [97]. MG is an autoimmune disease caused mainly by autoantibodies against acetylcholine receptor (AChR), although other autoantibodies can result in MG. These antibodies reduce nerve impulses, resulting in the typical fatigue-induced weakness observed in the muscles controlling eye movements, swallowing, breathing, posture, and limb movements [98].

Patients with MG will experience a more severe course of infection with COVID-19 and poorer outcomes when compared to non-MG patients. This unfortunate statistic results from a comprehensive pathomechanical interaction between COVID-19 and MG, which includes dysregulation of the immune system, immunocompromised state of MG patients due to immunosuppressive treatment, weakness of respiratory muscles, and higher frequency of respiratory failure due to infections or thromboembolic respiratory events. The relationship between COVID-19 and MG is bi-directional, meaning that COVID-19 can also cause exacerbations of MG due to molecular mimicry and induction of the T-cell dysregulation resulting in autoimmune reactions, and the secretion of pro-inflammatory cytokines leading to cytokine storm, acute respiratory distress syndrome (ARDS) [99].

Few interesting pharmacological correlations between COVID-19 and MG were described in the literature. Azithromycin and hydroxychloroquine, proposed for COVID-19 treatment, may potentially increase the risk of myasthenic crisis. Although there is no direct evidence linking the administration of these drugs to such crises in mild COVID-19 cases, caution is advised by local experts [100,101].

Studies have shown no direct correlation between COVID-19 severity and steroid use, with some even suggesting a protective effect of steroids in MG patients, resulting in lower mortality rates and reduced ventilator usage [102,103]. Other studies showed that older age and long-term steroid use before COVID-19 infection in MG patients were associated with a more severe infection course [104]. MG patients receiving rituximab, a CD-20 monoclonal antibody, were found to be at an increased risk of COVID-19-related death due to a lack of antibody formation against the virus [104].

Effects of COVID-19 treatments on neurological pathologies

Parkinsonism and COVID-19 Treatment

PD patients with COVID-19 had further exacerbation of already underlying disease possibly linked with secondary neurodegeneration [105]. Some authors also suggested that another possible reason for the major acceleration of the ongoing course of disease progression was due to the involvement of dopamine in COVID-19 infection, mainly amantadine and alpha-synuclein. Most of the patients with PD were on oral carbidopa-levodopa. This was the most common drug for which dose modification was needed [106]. Older advanced PD patients may represent a particularly vulnerable population, as respiratory muscle rigidity, as well as impairment of cough reflex alongside preexisting dyspnea, may lead to increasing severity of COVID-19 [107]. In addition, there are indirect possible effects, such as the impact of stress, self-isolation, and anxiety as well as the consequence of prolonged immobility because of the lockdown. Another class of drug that showed frequent interaction with COVID-19 treatment, mainly antitussives, was monoamine oxidase B (MAO-B) inhibitors [108]. In patients who had high sputum production and excessive shortness of breath, higher doses of antitussives were required, leading to the complete ineffectiveness of MAO-B inhibitors. Interactions with other classes of drugs including DDC inhibitors, dopamine agonists, and COMT inhibitors were hard to access as not much data is available. There is some data available that state that PD patients on amantadine were needed for dose modification. There are also certain publications that say the contrary, i.e., that patients who were on amantadine were less prone to the development of COVID-19 protein [109]. However, the above result warrants further studies to establish a clear-cut relation.

Encephalitis

It is also seen that high doses of intravenous immunoglobulin therapy (IVIG) (> 15gm/day) given in the early stage of COVID-19 infection (admission <= 7 days) have shown a remarkable decrease in mortality in critical patients [110]. It is observed that despite the reduction in mortality, the treatment has limited outcomes [110]. The data shows that in patients who recover from encephalitis, neurological dysfunction may persist and one-third of the patients at discharge had cognitive impairment. Along with viral encephalitis, it is noticed in a recent literature review that there was evidence of auto-immune encephalitis triggered by COVID-19. It is worth noting that this capacity of the SARS-Cov-2 virus in any form either as infection or through vaccination has been documented where acute encephalitis was seen in a case report as well as a literature review which summarized the prevalence of acute encephalitis after the first dose of COVID vaccination (mRNA-1273). After receiving vaccination, patients presented with symptoms of altered mental status, and involuntary movements suggestive of encephalitis like picture. The treatment was the same as mentioned above (prednisolone) along with anti-convulsants [110].

MS

The main concern in people with MS is that, firstly, their treatment consists of immunomodulators which decrease natural immune responses to infections and, secondly, hospitalization for monitoring of the administered treatment makes the patients susceptible to acquiring COVID-19 infection. During the pandemic of SARS-CoV-2, due to the increased exposure to this virus along with altered immune responses, practitioners had to optimize the treatment plan which took into consideration the risks and benefits of giving patients MS treatment to making them prone to acquire SARS-CoV-2. Studies have shown that a certain component of the immune system is affected by the COVID-19 virus compared to the immunomodulators used in MS which affect the functioning of different sets of immune cells [111]. However, more information is needed about the functioning of the immune system affected by the drugs and the ones affected by COVID-19 in order to understand the potential risk to people with MS. The dilemma that this poses is that we have to choose between “poorly treated MS for prevention from COVID-19 infection” and “COVID-19 infection while on MS treatment”. In order to help with this, studies have categorized drugs for MS on the basis of the severity of the risk of acquiring COVID-19, developing its complications, and worsening of MS symptoms. These categories are low-, intermediate-, and high-risk drugs which are differentiated on the basis of the safety of MS patients. For example, the low-risk category involves interferon-beta which is both safe to start when first diagnosed and continue if already started [111].

Studies have shown that natalizumab is the preferred drug of choice in patients with MS and is also a high efficacy agent as it is rapidly reversible (I/c/o plasma exchange) and does not inhibit migration of immune cells into the lymphoid tissue which keeps novel response intact. Therefore, it does not alter immunity and is safe to use for the treatment of COVID-19.

Another drug that has been studied is ocrelizumab. It is seen that patients who are treated with ocrelizumab have mild-to-moderate severity of COVID-19 infection, mostly not requiring hospitalization [112].

*GBS*

Although the occurrence of GBS is quite rare, 436 cases were reported post-COVID. The most common type of GBS was found to be AIDP [113]. It was particularly noticed that the severity of COVID-19 was directly proportional to the number of GBS cases. This conclusion came from noticing an increased number of GBS cases in ICU-admitted patients. However, improvement in GBS was quite commendable. The mechanism of action (MOA) for the manifestation of GBS is under evaluation. Research is needed for the para-infectious and post-infectious course of COVID-19 [113].

Treatment options available for GBS are IVIG - 2g/kg over five days (MOA: immuno-modulating); plasma exchange - five sessions of volume exchange (MOA: removing pathogenic antibodies, humoral mediators, and complement proteins). No difference has been recorded between these two treatment modalities in terms of efficacy and safety [114].

Stroke

Post-COVID-19 stroke has been studied extensively and is quite worrisome as the number of cases reported has increased immensely. The most common age group affected by this is the elderly (mean age = 72 years). Acute ischemic stroke was most recorded 10 days after the first symptom of COVID-19. A study of 221 people with COVID-19 was done out of which 11 had stroke. Out of 11, five people had large artery stroke, three had small artery stroke and three had cardio-embolic stroke [115]. The treatments given to these 11 patients were as follows: six patients were given anti-platelet treatment with aspirin/clupidogrel. The mortality noted with this group of patients was 50%. The other five patients received anticoagulant treatment (enoxaparin). The mortality was reduced to 20%, indicating the obvious safety of anticoagulants in post-COVID acute ischemic stroke [115].

Drugs and therapeutics

General Therapeutics of COVID-19

Since the beginning of the pandemic, multiple treatment options for SARS-CoV-2 have been used, although the natural process of viral mutation has raised different variants, such as changing the treatment options and vaccine efficacy and effectiveness [116]. The COVID-19 therapeutics can be divided into two groups: the antivirals and the immunomodulators [116].

Recent studies have shown that current antiviral treatments for COVID-19 do not change efficacy compared to the new variants [116]. The mechanisms of these drugs can be separated into virus entry to the host cells and replication suppression inside the cells. Within the entry-block mechanism group, a new therapeutic prospect is the ACE2-targeted drugs (e.g., colchicine, ursodeoxycholic acid (UDCA)), which have been shown to reduce viral load and replication by decreasing the ACE2 activation in the lungs and other organs but need further development and research [117]. This research has found that inhaled administration of these prospective drugs is the best option because it reduces the necessary dose and side effects [117]. Furthermore, antivirals that block steps of viral replication within the cell (e.g., chloroquine, griffithsin, and nafamostat), virus replication, transcription, and translation (e.g., remdesivir, favipiravir, ribavirin, bananin, and 5-hydroxy chromone), inhibitors of endosome maturation (e.g., hydroxychloroquine, apilimod, colchicine, and vinorelbine), and release of the viral genome (e.g., cinanserin, disulfiram) have been used for SARS-CoV-2 treatment, with variety of efficacy for lowering the viral load and replication [117].

Despite the great worldwide effect of vaccination in reducing the propagation of SARS-CoV-2, some side effects that range from mild to severe have been reported. A spectrum of serious unexpected neurological complications has been reported, such as cerebral venous sinus thrombosis, Bell's palsy, post-vaccine encephalitis, acute ischemic stroke, transverse myelitis, ADEM, and GBS, among others. These complications could occur after molecular mimicking by the vaccine biomolecules with host molecules. However, these findings need further research for causality and are a topic of great controversy [118].

Therapeutic approaches for neurological manifestations of COVID-19

Much research is being done on the effects and outcomes of COVID-19 and its neurological manifestations, and much of that research today includes the therapeutic approaches to the interactions of certain neurological diseases and COVID-19. Here, we focus on the therapeutics connected to stroke, encephalitis, GBS, PD, MS, neuropathic pain, and MG. Many neurological therapies for patients who were hospitalized for COVID-19 include using steroid treatment such as prednisone therapy or immunoglobin IV therapy [119].

Stroke

During the COVID-19 pandemic, many patients who had been infected by SARS-CoV-2 had an increased risk for thrombotic complications such as higher chances of having a stroke [120]. Some of the reasons for this included delayed treatment for COVID-19 and venous thrombosis after vaccination of the adenovirus vector [119]. Incidence of stroke in patients with COVID-19 shows that treatment of the virus early on allowed for lower rates of stroke in patients as complications. Data was analyzed from 77 random control trials and 38,732 COVID-19 patients showing that the stroke incidence is only about 0.17%. The therapeutics for the inpatients in this study included the neutralizing antibody bamlanivimab [120].

There are no specific drugs to treat stroke in COVID-19 patients other than treating the virus to prevent any risk of stroke occurring. Moreover, the use of ACE or angiotensin-receptor blockers is highly suggested for hypertensive patients who are at risk for stroke. Neurorehabilitation and mirror therapies were forms of physical therapy for patients who had strokes to use in the pandemic [121]. When using reperfusion therapies, it was found that there was no significant difference in stroke patients before versus after being infected by COVID-19 in a cohort study of 545 thrombolyzed patients in which 101 had COVID-19 [122]. Because intra-cranial hemorrhages are a major reason for strokes, it is recommended that COVID-19 patients should not be taking therapeutic anticoagulation or mechanical ventilation to prevent the incidence of ICH. Moreover, COVID-19 patients with venous thromboembolisms should receive low molecular weight heparin or unfractionated heparin treatments as thromboprophylaxis [123,124].

Encephalitis

While COVID-19 has caused some chronic issues, many complications include acute diseases such as encephalitis. Several therapeutics have been used for encephalitis alone, but when patients are affected by COVID-19, some case studies have been done to compare the treatments. A case study where a 39-year-old man who came into the hospital with a positive nasal swab for SARS-CoV-2 also came in with symptoms of acute demyelinating encephalomyelitis that was mimicking encephalitis and was successfully treated with immunoglobulin therapy along with cytokine blockade with intravenous tocilizumab. As tocilizumab is an IL6-receptor antagonist, this may have been related to a host inflammatory response [125]. Another such case study was where a 60-year-old patient with severe SARS-CoV-2 infection developed encephalitis, which was treated with corticosteroids. He responded very well to the therapeutic effects of the high-dose corticosteroid administration due to a possible inflammatory-mediated brain involvement related to COVID-19 [126].

Encephalitis, being a life-threatening COVID-19 neurological syndrome, can vary in individual patients. In another case study, three out of five patients showed dramatic improvement in their neurological impairment after beginning immunotherapy, which allowed for bettering of their functional communication. The other two patients showed no improvement after this immunotherapy, combining corticosteroid infusions and TPE with albumin. However, after further case studies, the findings support that immunotherapy with this combination therapy is effective in treating severe COVID-19-related encephalitis [127]. Another beneficial treatment for encephalitis that arose in patients with COVID-19 is plasma exchange. Patients with encephalitis have improved after immuno-therapies such as IV methylprednisolone and IVIG but moreso showed rapid improvement in three out of five patients after therapeutic plasma exchange [128]. High-dose steroid therapy is also most favorable in treating autoimmune encephalitis that arises due to COVID-19 and their vaccines [129].

*GBS*

GBS syndrome was one of the very first documented autoimmune neurological diseases triggered by COVID-19. Some research shows the beneficial effect of anti-complement therapies on this neurological complication of COVID-19 as well as the associated respiratory distress syndrome [130]. In a few studies, the therapeutic response to therapies, including IVIg and/or plasma exchange in GBS associated with COVID-19, was more favorable than the response to corticosteroids. However, with opposing results in other studies of suboptimal therapeutic responses in patients with GBS, more data and studies are needed to confirm if corticosteroids are concurrently beneficial [131].

Post-COVID-19, another unique case of GBS showed that IVIg caused treatment-related fluctuation. A 35-year-old male patient developed GBS following the COVID-19 infection and was given this immunoglobulin therapy when he had fluctuations and was put on ventilator support, but repeat IVIg therapy caused him to have a complete recovery. The mechanism of action of immunoglobulins is complement inactivation, antibody neutralization, inhibition of cytokines, and saturation of Fc receptors on macrophages [132]. In another study, IVIg and plasma exchange are shown to have the same efficacy level in GBS syndrome, but plasma exchange is considered the gold standard for GBS for its speed of action [133].

As seen in multiple other studies and articles, the best therapeutic for GBS related to COVID-19 is the standard IVIg therapy and plasma exchange treatment. The difference between regular treatment before and after being affected by COVID-19 was not drastic, and patients should continue to follow the standard therapeutics of any GBS patient [134]. GBS, being an inflammatory polyradiculoneuropathy, is associated with numerous viral infections. After a single IVIg course, this uncommon condition was improved in most cases in eight weeks [135].

PD

Patients with PD have had a rough time coping with the COVID-19 pandemic due to the nature of this disease. PD affects the aged population, more commonly above the age of 60 years, and has a higher risk of having compromised immunity with COVID-19. COVID-19 alone has created a common drug list, including remdesivir, favipiravir, chloroquine, hydroxychloroquine, azithromycin, amantadine, and monoclonal antibodies. PD has its own preferred drugs for therapy, including levodopa, dopamine agonists, monoamine oxidase inhibitors, and so on. Amantadine has antiviral properties that target both the COVID-19 virus and PD [136]. Because of the nature and chronicity of PD, there are very few therapeutic options. For some subjects with severe COVID-19 symptoms, increased levodopa and non-oral dopaminergic therapies aided the patients in the course of the disease [137]. This motor neurodegenerative disease that involves several metabolic and psychological ailments along with hyposmia is seen in COVID-19 patients [138].

MS

MS patients are a special group of interest when having COVID-19. Many disease-modifying therapies (DMT) drugs are used for MS, with different mechanisms of action, T and B cell depletion, immunomodulators, and immune cell trafficking modulators, all leading to changes in the immune response. There are opposite ideas whereas the immunosuppressor drugs used for MS could be related to an increased risk for severe SARS-CoV-2, but it is found that MS patients respond very similar to the general population, and the most relevant risk factors among MS patients for severe SARS-CoV-2 are high disability and a progressive course of MS [139]. There is no reported relationship between SARS-CoV-2 infection and a higher risk of MS relapses [140].

In an Italian report of 1354 patients with MS and confirmed SARS-CoV-2 infection, the symptoms were similar to those without MS, such as fever, fatigue, cough, dyspnea, anosmia, and ageusia. However, the treatment for MS with the B cell-depleting anti-CD20 therapies (rituximab, ocrelizumab, ofatumumab, and ublituximab) [141], was associated with an increase in SARS-CoV-2 general symptoms, neurological disorders (weakness, fatigue, asthenia, and cognitive symptoms), conjunctivitis, and rash [142]. Recent data shows that patients on rituximab have a fourfold increased risk of SARS-CoV-2 hospitalizations [140,143]. On the other hand, they reported a protective role with the use of teriflunomide, natalizumab, and fingolimod, reducing the shortness of breath, tachycardia, and chest pain, in patients with SARS-CoV-2 infection [142]. Some MS therapies, such as beta-interferons, fumarates, and sphingosine-1-phosphate (S1P), don’t seem to modify the course of COVID-19 infection. Therefore, it is not recommended that DMT be stopped in patients with MS and SARS-CoV-2 [140].

The relationship between COVID-19 vaccines and MS has been widely investigated. Symptoms after vaccination in patients with MS are similar to those in the general population, such as injection-site pain, headaches, and fatigue. Furthermore, MS patients under anti-CD20 therapies or fingolimod are found to have weak humoral immune response after two weeks of COVID-19 vaccination. However, vaccination is still recommended for MS patients with three initial and two subsequent doses [140,144].

Neuropathic Pain

Long COVID-19 pain can have a broad spectrum of origins, vary among the population, and cannot be treated the same way for every individual. Instead, personalized treatment should be used for every type of pain, as it can be nociceptive, neuropathic, and neoplastic [145]. Neuropathic pain is a neurological condition that can be found in long-term COVID-19, as a result of a lesion in the somatosensory central or peripheral nervous system direct from COVID-19 infection or as a result of another COVID-19 neurological complication such as stroke and MS [146]. Up to 2.3% of hospitalized COVID-19 patients may suffer neuropathic pain; other neurological complications can be a risk factor for neuropathic pain, such as stroke, myelitis, and GBS [147]. The development of post-herpetic neuralgia, trigeminal neuralgia, or brachial plexopathy are well-described examples of this neuropathic long-COVID-19 pain. Importantly, the real prevalence of these conditions measured by objective tests remains unclear, and further research has to be conducted [145]. Neuropathic pain as a COVID-19 sequela has been treated with several drug options, including gabapentin, serotonin-norepinephrine reuptake inhibitors, tricyclic antidepressants, tramadol, strong opioids (morphine, oxycodone, and methadone), among others [148,149]. However, there is no FDA-approved drug therapy for chronic COVID-19 pain, nor has any randomized, controlled, clinical trial that evaluates a particular treatment for chronic pain related to COVID-19 been published, and therefore, any type of chronic pain as a result of COVID-19 infection should be addressed by the standards of pain therapy [150,151].

MG

COVID-19 treatment may worsen MG symptoms; drugs such as hydroxychloroquine and azithromycin can cause MG exacerbations [152]. Recent research has explored the effect of different COVID-19 drugs on MG. Colchicine could rarely develop myopathy in patients with MG as the only reported effect. Other drugs, such as antivirals (remdesivir, lopinavir, ritonavir, and favipiravir), are safe to use, and no effects have been reported; others, such as tocilizumab and eculizumab, are not only safe to use but have shown positive effects on MG [153]. Vaccination against COVID-19 has shown that it is safe and does not carry complications or exacerbations in MG patients. The side effects are similar to those of the general population, like fatigue and mild pain in the injection site [153].

Because of their immunocompromised state, MG patients are at greater risk of COVID-19 infection in 20-50% of cases. Furthermore, treatment options used for MG in relation to COVID-19 infection have different outcomes; research has found that using prednisone alone as a treatment for MG before or during COVID-19 infection increases the mortality rate of the infection [154]. The use of rituximab as a treatment for MG increases the severity of COVID-19 disease. Combining prednisone with other drugs (e.g., intravenous immunoglobulin and plasma exchange) could be protective, reducing the MG severity during COVID-19 [154]; acetylcholine esterase inhibitors may have positive effects on COVID-19 and should not be discontinued; others have not enough data to conclude their effects on COVID-19 but are probably safe to use; such drugs are mycophenolate mofetil, azathioprine, cyclosporine, and methotrexate [153].

ACE2 as a therapeutic target

ACE2 could also be considered a therapeutic target for SARS-CoV-2 infection. Nevertheless, more research on drugs is needed to clarify side effects and safety [155]. Recent research has found that colchicine can reduce the expression of ACE2 receptors in lung cells by inhibiting the transcription factor SP1. Colchicine in this study showed a markedly severity reduction in histopathological lung and renal damage in Syrian hamsters [156]. The ursodeoxycholic acid (UDCA) also downregulates ACE2 expression by suppressing the farnesoid X receptor (FXR) pathway, decreasing the susceptibility to SARS-CoV-2 infection [157,158]. A promising drug that has been researched using the SARS-CoV-2 interactions with ACE2 is methotrexate, which can be used as a preventive measure. Kim et al. (2024) explored methotrexate as a binding inhibitor for COVID-19 and found that it can bind to SARS-CoV-2, avoiding its binding to ACE2 and, therefore the viral infection. They demonstrate that methotrexate also binds to the Alpha, Delta, and Omicron variants, assuring a wide therapeutic effect for the actual COVID-19 variants. Besides methotrexate's range of side effects, this interaction needs a minimum dose that can be delivered straight to the lungs via inhalers, reducing the potential side effects. Further research is needed to determine the appropriate dose [159]. In recent research, it was found that an immunological molecule related to ACE2 could prevent the COVID-19 infection; in that study, they used biomolecular engineering, combining ACE2 with Fc, creating an antibody-like molecule that would attach to the virus and prevent the SARS-CoV-2 infection. This newly explored therapy could create anti-drug antibodies, which affect safety and create a wide range of side effects [160].

Post-vaccination neurological complications

While severe neurological complications related to COVID-19 vaccinations are a rare phenomenon, mild and distinct symptoms can occur post-vaccination. Potential problems such as GBS, cerebral venous thrombosis, or seizures/optic neuritis are likely, but those are rare and usually experience a good recovery with prompt medical treatment. The ChAdOx1nCoV-19 vaccine is linked with a slightly increased risk of neurological disorders, such as GBS and Bell’s palsy, but these events are much still less frequent than the risk from COVID-19 infection itself [161]. Such case reports by victims are one of the factors adding to people’s fears about the diseases being responsible for catastrophic complications such as deaths. Here is the evidence that this confirms the need for efficient communication between the medical team and the general public regarding the highs and lows of COVID-19 vaccines and the hazards of serious adverse reactions, which are very rare [162]. One separate NMOSD case has been reported among non-COVID-19 vaccine recipients, which indicates that the vaccinations may still be an existent risk for people with autoimmune abnormalities, although such complications are very rare [163]. This substantial investigation probed a spectrum of neurological symptoms that showed up after receiving the COVID-19 vaccine in Milan, and instances of tremors, disturbances in sleep, cramps, twitches, and headaches were noticed, among others. [164]. A thorough examination was done in which the researcher studied neuropsychiatric syndromes such as body pains, paresthesia, and even severe cases like urinary retention and facial droop with different vaccination types, thereby confirming once more the rarity but the possibility of such reactions [165]. Studies conducted mainly in the form of case series have revealed an increase in the cases of GBS and severe conditions following the first dose of the Oxford-AstraZeneca (ChAdOx1nCoV-19) vaccine. In some cases, like in the Oxford-AstraZeneca vaccine, the risk is more in the form of serious reactions [166]. A case study done in Saudi Arabia to investigate life-threatening complications such as cerebral venous thrombosis and GBS indicated the rarity of such events while the significance of this was noted [167]. Electroencephalogram control has indicated neural profiles after the injection, suggesting slight to nearly life-threatening effects that have to be considered in clinical evaluations [168].

## Conclusions

The relationship between the COVID-19 virus and the nervous system is a complex and critical aspect of the pandemic, with significant implications for public health, patient care, and medical research. The diverse range of neurological disorders associated with COVID-19, including the involvement of the central nervous system through various routes and mechanisms, highlights the intricate pathogenesis of SARS-CoV-2 infection and the remarkable diversity of the syndrome. Unlike many other viruses that primarily cause acute manifestations such as stroke and encephalitis, COVID-19 can also exacerbate chronic conditions such as Parkinson's disease and multiple sclerosis. These unique cases present challenges in terms of diagnosis, treatment, and long-term management. This review underscores the importance of studying the neuroinvasive properties of SARS-CoV-2, the defining role of ACE2 in their manifestations, and the systemic inflammation and immune dysfunction that are at the forefront of neuropathogenesis. These skills are crucial for developing precise treatment mechanisms, improving patient response, and enhancing healthcare techniques in COVID-19 cases. As the pandemic continues to evolve, ongoing research is essential to understanding the long-term neurological complications of COVID-19, identifying early detection and prognosis biomarkers, and discovering novel therapeutic pathways. The collaboration of endocrinology, virology, immunology, and pharmacology, among other disciplines, will play a critical role in comprehending and managing brain conditions linked to the COVID-19 pandemic. Despite the challenges, the pandemic has also brought new opportunities for research and innovation in the quest to restrict and even cure neurological diseases.
